# Validation of the PHQ-9 depression scale in Ethiopian cancer patients attending the oncology clinic at Tikur Anbessa specialized hospital

**DOI:** 10.1186/s12888-020-02850-3

**Published:** 2020-09-10

**Authors:** Mikyas Degefa, Benyam Dubale, Fikirte Bayouh, Biniyam Ayele, Yared Zewde

**Affiliations:** 1 Amanuel Mental Specialized Hospital, Addis Ababa, Ethiopia; 2 Department of Pyschiatry, College of Health Science, Addis Ababa University, Addis Ababa, Ethiopia; 3 Department of Neurology, College of Health Science, Addis Ababa University, Addis Ababa, Ethiopia

**Keywords:** Validation, Depression, Patient Health Questionnaire-9 (PHQ-9), Cancer, Ethiopia

## Abstract

**Background:**

Although depression is highly prevalent among cancer patients, it is often underdiagnosed and poorly managed particularly in developing nations. These shortcomings can have substantial adverse effects not only on the disease prognosis but also on patients’ quality of life. The Patient Health Questionnaire-9 is a widely used depression screening tool but it has not been validated among patients with chronic illnesses such as cancer in Ethiopia. We aim to validate the PHQ-9 among Ethiopian cancer patients in an outpatient setting.

**Methods:**

A cross-sectional study was conducted among cancer patients attending the oncology clinic at Tikur Anbessa Specialized Hospital (TASH). We assessed criterion validity and performance of the PHQ-9 test against the gold standard Mini-International Neuropsychiatric Interview (MINI) diagnostic tool among patients with cancer. The MINI was administered by psychiatric nurses who were blind to the initial PHQ-9 screening tool.

**Results:**

A total of 163 patients completed the 2 stages of a diagnostic interview in the study. The majority (64%) of the participants were women, the mean age was 46 (13.5) years. Using the gold standard MINI test the prevalence of Major Depressive Episode (MDE) was 15%. The internal consistency (Cronbach’s α) for PHQ-9 was 0.78 suggesting good (acceptable) internal consistency for the reliability of the test scores. When the total PHQ-9 score was used to identify cases of MDE, the Area under the Curve (AUC) was 0.93 (95% confidence interval [CI], 0.88–0.97) on Receiver Operating Characteristic (ROC) analysis. This shows evidence for the excellent discriminating power of the PHQ-9 between cases and non-cases of MDE. At cutoff point ≥4, the PHQ-9 had a sensitivity of 88% and specificity of 78.1% on the ROC curve to detect MDE.

**Conclusion:**

PHQ-9 is a reliable and valid instrument to detect MDE among individuals with chronic conditions such as cancer patients in outpatient settings and it can be used in resource-limited settings for early diagnosis and proper therapy of such patients.

## Background

Cancer is a chronic medical illness with 18.1 million new cases and 9.6 million deaths only in 2018. Cancer is also an emerging public health issue in Africa, with estimates of 811,200 new cases and 533,800 cancer deaths in the same year. In Ethiopia, the annual incidence and mortality from all cancer types were 67,500 and 50,000 cases respectively [1]. Depression is one of the most common psychiatric comorbidities following the diagnosis of cancer. The prevalence of depression among patients with cancer ranged widely from 3% up to 50% depending on the method of ascertaining depression, study population concerning to cancer type, stage, treatment modality, and the use of different instruments. However most estimates for the prevalence of depression falling between 10 and 25% [2]. Studies show that clinicians working in cancer services have recognized that depression is often undiagnosed and untreated, and these shortcomings can have substantial effects, not only on patients’ quality of life but also on their acceptance of cancer treatment [3, 4] This co-morbidity of chronic medical condition with depression is a public health concern due to its negative effects on the course of the illness and its impact on overall prognosis [5, 6].

Detecting depression is often difficult in cancer patients because of overlapping symptoms such as fatigue, loss of appetite, sleep disturbance, and also the effects of cancer treatment have been thought to have a confounding effect on the assessment of depression [7, 8]. In addition to this, stigma, lack of healthcare providers trained in mental health, and paucity of validated screening and diagnostic tools also contribute to the low level of diagnosis and treatment of depression, particularly in Low and Middle- Income Countries (LMIC) [9–11]. Screening instruments such as the PHQ-9 have been designed to detect MDE according to the Diagnostic and Statistical Manual of Mental Disorders-IV text revision (DSM-IV-TR). This instrument is free, takes a brief time to administer, and simple to score. This makes it ideal for use in clinical settings where administering comprehensive structured or semi-structured screening instruments can be difficult due to busy clinics and few health professionals [12]. In two different studies done in Kenya among patients with chronic medical conditions, PHQ-9 was shown to be a reliable instrument for the detection of depression [13, 14].

In Ethiopia, two prior studies concluded that PHQ-9 was a valid and reliable instrument for detection of major depressive disorders among patients in outpatient settings and rural communities, while the latter study also emphasized the need for further study in the utility of the screening tool in clinical settings [15, 16]. Structured diagnostic interview tools are available for diagnosis of depression in patients with chronic medical conditions including the MINI which allows diagnosis of depression according to DSM-IV and ICD-10 criteria [17].

The objective of this study is to evaluate the criterion validity of the PHQ-9 for detecting depression among patients with cancer attending the outpatient oncology clinic at Tikur Anbessa Specialized Hospital.

## Methods

### Study setting and period

Tikur Anbessa Specialized Hospital (TASH) is the largest and oldest referral hospital in Ethiopia which provides comprehensive multidisciplinary medical service for the nation including oncology service. The oncology care in TASH is comprised of inpatient and outpatient services including the only radiotherapy service in the country. It provides services for patients referred from different parts of Ethiopia. The data were collected between August and September 2016.

### Study population

The study population was all adult patients with a diagnosis of cancer attending the outpatient oncology services in TASH.

### Sample size

Using a convenient sampling technique, we enrolled all consecutive patients who came for follow up during the study period. The sample size was determined using a formula for calculating sensitivity and specificity for single tests [18]. With sensitivity at 85% and a prevalence of 30%, the total number of patients expected in our study was 163. This allowed the estimated sensitivity to be within the confidence limits of 80 and 90%. Patients who were younger than 18 years of age, those who were in severe distress requiring emergency care, and those who failed to communicate in Amharic (the federal working language of Ethiopia) were excluded.

### Study design

We used a hospital-based cross-sectional study design. Participants’ socio-demographic characteristics including age, educational level, marital status, occupation, and residential place were documented. Types of cancer, time of diagnosis, stage of cancer, treatment history, and duration of the illness were retrieved from the chart and the participants. The study questioners including the PHQ-9 tool were administered by two oncology nurses. The MINI was administered by two psychiatric nurses. All data collectors received in-depth training on the study instruments, ethical conduct of research, and data collection techniques for 3 days by a qualified independent mental health researcher.

### Screening test

The PHQ-9 comprises nine items that can be scored from 0 (not at all) to 3 (nearly every day) and the total score ranges from 0 to 27 to measure depression severity [19, 20]. So far only one study has been conducted in Ethiopia using the Amharic translated version of PHQ-9 in a medical outpatient setting for detection of depression [21].

### Diagnostic criterion measure of depression

The MINI is a gold standard brief assessment tool that allows the diagnosis of depression according to DSM-IV and ICD-10 criteria [22]. It is modularized and each major diagnostic condition is represented by a module. For this validation study, the module on Major Depressive Episode was used.

### Data collection and management

#### Two-stage sample selection

After getting a written informed consent, socio-demographic and clinical data together with the test assessment (PHQ-9) score for each participant were collected by two trained oncology nurses. Later on the same day, each patient was again re-assessed using the gold standard assessment MINI by two qualified psychiatric nurses. The data collectors readout and elaborated all the questions for the illiterate participants. The psychiatric nurses who were conducting the criterion assessment interviews were blinded to the results of the PHQ-9 and vice versa.

### Data analysis

Data were analyzed using the Statistical Package for Social Sciences version 20.0 software package (SPSS Inc., Chicago, IL, USA). Initially, one case was excluded due to a missing value in the PHQ-9 data and then a sample of 162 cases were categorized into cases of MDE and non-cases based on the MINI assessment to determine the validity of the instrument.

The sensitivity, specificity, positive likelihood ratio, and negative likelihood ratio were calculated to determine different cutoff scores for PHQ-9. Receiver Operating Characteristic (ROC) curve was used to identify optimal balance between sensitivity and specificity for the determination of the best PHQ-9 cutoff score for the diagnosis of major depression. Youden index (sensitivity+ specificity-1) was converted into a percentage and was used as an additional metric for cutoff determination, where measure above 50% was considered as acceptable values of diagnostic accuracy [23].

The area under the curve (AUC) was used to address the performance of the test. Reliability related to internal consistency was measured by Cronbach’s alpha coefficient (Cronbach’s α).

## Results

### Socio-demographic characteristics

A total of 163 patients completed the two-stage process of the diagnostic interview. The mean age of the participants was 46 (+ 13.5) years and 64% of them were females. More than two-thirds (73%) of our participants were educated from which 37% earned a college degree. Two-thirds (66.3%) of the participants were married. One in three (31.3%) described themselves as housewives and 70% were from rural areas (Table 1).

**Table 1:** Description of the socio-demographic characteristics of participants

	Frequency	Percent (%)
Age in years		
18–40	68	42
41–60	73	45
> 60	22	13
Gender		
Male	59	36
Female	104	64
Literacy		
Literate	118	73
Illiterate	43	27
Level of Education		
Elementary education	34	29
High school education	29	25
College & above	44	37
Informal education	7	6
No education	4	3
Marital status		
Married	108	66.3
Single	19	11.7
Divorced	9	5.5
Widowed	18	11.0
Residence place		
Urban	48	29.4
Rural	115	70.6
Occupation		
Housewives	51	31.3
Farmer	26	16
Merchant	25	15.3
Government	28	28.2
Student	6	3.7
Daily laborer	2	1.2
Unemployed	3	1.8
Other	4	2.5

### Distribution of cancer-related clinical characteristics

The most frequent type of cancer identified was breast cancer 28.7% followed by skin cancer (16%) and gastrointestinal cancer (12%). According to TNM classification more than half (53%) of the patients were at stage II and followed by stage III at 27% and stage I at 18%. At the time of screening, almost half (47%) were receiving chemotherapy only, while 29% were receiving both treatments. The majority of the participants (81%) got their diagnosis between 1 and 5 years while those diagnosed in the past 1 year were 5.5% (*n* = 9) (Table 2).

**Table 2:** Frequency distribution of cancer-related clinical characteristics of study participants

Cancer-related variables	Frequency (N)	Percent (%)
Types of cancer		
Breast	46	28.7%
Skin	26	16%
Gastrointestinal	22	13.5%
Cervical	20	12.2%
Oropharyngeal	15	9.1%
Prostate	9	5.5%
Others	25	15%
Stages of cancer		
Stage I	26	17.8%
Stage II	70	53.4%
Stage III	40	27.4%
Stage IV	2	1.4%
Treatment		
Chemotherapy only	77	47.2%
Radiotherapy only	33	20.2%
Chemotherapy & radiotherapy only	48	29.4%
Chemotherapy & surgery only	2	1.2%
Radiotherapy & surgery only	1	0.6%
Not on treatment	2	1.2%
Duration of Illness		
< 1 year	9	5.5%
Between 1 year and 5 years	132	81%
> 5 years	22	13.5%

### Depression among cancer patients

The prevalence of MDE among cancer patients in this study using the gold standard MINI was 15.3%(*n* = 25). A quarter of cervical cancer patients were diagnosed with MDE which is the highest compared to other types of cancer. Nineteen percent (*n* = 15) of patients with stage II cancer and 24% (*n* = 8) of patients receiving only radiotherapy treatment were diagnosed with MDE (Table 3). The mean score of PHQ-9 was 2.81 and on each item, the mean score ranged from 0.06 (suicidal ideation) to 0.56 (loss of energy) (Table 4).

**Table 3:** Prevalence of depression using MINI among different cancer types, stage, and different treatment

Types of cancer	Total (N)	MDE (N)	Percent (%) MDE
Breast	47	6	13%
Skin	26	4	15%
Gastrointestinal	22	2	9%
Cervical	20	5	25%
Oropharyngeal	15	2	13%
Prostate	9	1	11%
Others	24	5	21%
Total	163	25	15.3%
Cancer stage			
Stage I	26	3	11.5%
Stage II	78	15	19%
Stage III	40	3	7.5%
Stage IV	2	1	50%
Total	146	22	15.3%
Treatment			
Chemotherapy only	77	7	9%
Radiotherapy only	33	8	24%
Chemo & radiotherapy only	48	7	14.6%
Chemotherapy & surgery only	2	0	0%
Radiotherapy & surgery	1	1	100%
No treatment	2	2	100%
Total	163	25	15.3%

**Table 4:** Mean scores of PHQ-9 Items

PHQ Items	Mean	Standard Deviation
Loss of interest	.38	.68
Feeling depressed	.42	.70
Sleep problems	.42	.71
Loss of energy	.56	.75
Appetite problems	.37	.69
Self-blame	.19	.50
Concentration problems	.28	.61
Agitation/retardation	.13	.37
Suicidal ideations	.06	.33
Sum score	2.81	5.37

### Reliability

The reliability coefficient, Cronbach’s α was 0.78 indicating acceptable internal consistency for the reliability of the PHQ-9 test scores.

### Criterion validity of the screening instruments against a gold standard

**Fig. 1:**
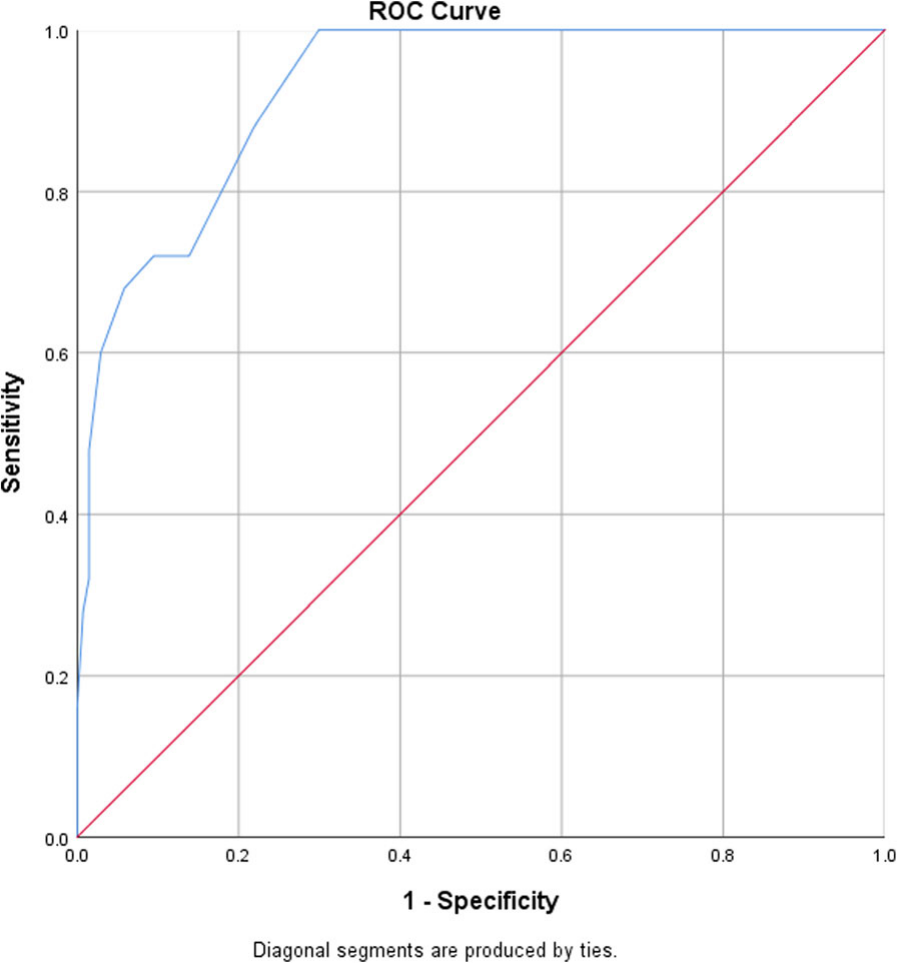
ROC curves for PHQ-9 against gold standard (MINI) diagnosis of MDE

After excluding one missing case, a total of 162 cases were analyzed to determine the criterion validity of PHQ-9 against the gold standard MINI. The area under the ROC curve was 0.93 (95% confidence interval [CI], 0.88–0.97) on analysis. According to this result, PHQ-9 showed an excellent discriminating power to differentiate between cases and non-cases of MDE. The detailed description of the ROC curve for PHQ-9 against the gold standard (MINI) is shown in Fig. 1. The optimal cutoff point with maximum sensitivity and without loss of significant specificity was ≥4; the PHQ-9 had a sensitivity of 88% and specificity of 78.1% on the ROC curve. At this cutoff score, a person testing positive for MDE is 4 times more likely than a person who doesn’t have MDE to be tested positive. Youden index also showed a higher value with 66.1%. The detailed description of the cutoff scores of PHQ-9 against the gold standard (MINI) is shown in Table 5.

**Table 5:** PHQ-9 cutoff scores against the gold standard of study participants

Optimal cutoff point	Sensitivity	Specificity	+LR	-LR	Correctly classified	Youden index
≥1	100%	47.4%	1.9	0	55.5%	47.4%
≥2	100%	58.4%	2.4	0	64.8%	58.4%
≥3	100%	70%	3.4	0	74.7%	70%
≥4	88%	78.1%	4	0.09	79.6%	66.1%
≥5	72%	86.1%	5.2	0.24	83.9%	58.1%
≥6	72%	90.5%	7.5	0.25	87.6%	62.5%
≥7	68%	94.1%	11.7	0.3	90.1%	62.1%
≥8	60%	97.1%	20.6	0.3	91.3%	57.1%
≥9	48%	98.5%	33.1	0.51	90.7%	46.5%
≥10	32%	98.5%	22	0.67	88.2%	30.5%
≥11	28%	99.2%	38.3	0.71	88.2%	27.2%
≥12	16%	100%	0	0.84	87%	16%
≥13	12%	100%	0	0.87	86.4%	12%
≥14	4%	100%	0	0.96	85.1%	4%

## Discussion

In patients with chronic conditions recognizing and managing depression is important due to its potential in improving medication adherence, reducing the progression of the disease, and improving quality of life [24]. In our study, the prevalence of depression in adult patients with cancer attending outpatient clinics using the gold standard was 15%. This is comparable with pooled mean prevalence ranging from 8 to 24% in a meta-analysis of 211 studies [25]. Another meta-analysis on 94 studies among cancer patients reported a pooled prevalence of depression at 16.3% [26]. This result demonstrates a high prevalence of depression among patients with a diagnosis of cancer and underscores the need for a brief reliable and valid instrument for better detection and improved quality care. In this study, PHQ-9 has acceptable internal consistency with Cronbach’s α 0.78. A similar finding (Cronbach’s α of 0.84) was reported in Germany [27]. When we turn to the case detection property of the instrument it’s found to be highly accurate with the area under the curve (AUC) of 0.93 (95% [CI], 0.88–0.97) on ROC analysis. This is evidence of an excellent discriminating power between cases and non-cases of MDE. Our result was comparable to a similar study on cancer patients attending an outpatient clinic with a ROC curve of 0.94 (95% confidence interval [CI], 0.93–0.95) [28].

The choice of the optimal cutoff score is always a tradeoff between sensitivity and specificity. A lower cutoff score makes the questionnaire very sensitive and inclusive, whereas a higher cutoff score will make it more specific at the cost of missing some cases [20]. Meta-analysis 18 studies and 7180 participants found the PHQ-9 with cutoff scores between 8 and 11 have acceptable screening properties for detecting depression [29]. In our study at a lower cutoff point of ≥4, the PHQ-9 had a sensitivity of 88% and specificity of 78.1%. With a similar cut off point, our study yielded a better sensitivity and specificity compared to study done at South Africa in chronic care patients with sensitivity 87% and specificity 63.3% [30]. A study on somatic symptoms in depression concluded that somatic symptoms were common but had less impact on the diagnosis of depression, rather the core depressive symptoms of depression were better predictors for the diagnosis [31]. In our study, the mean scores of PHQ-9 items showed a relatively similar distribution between core symptoms of depression and somatic symptoms.

The strength of this study is that it is one of a handful of studies to consider the validity of the PHQ-9 in patients with the diagnosis of cancer in sub-Saharan Africa, the first in Ethiopia, and also one the few studies to provide the prevalence of depression among cancer patients in the country. We also used instruments that were previously translated into the Amharic language and validated in different settings. Data collectors and psychiatric nurses were blinded to the results of criterion assessment and screening instruments. Limitations of the study include the relatively small sample size, inability to test psychometric properties, and the factorial structure of the PHQ-9. We did not perform a regression analysis for the assessment of expected mean scores to evaluate the PHQ-9 mean values for different cancer types and we were also unable to explain the low rates of surgery among patients with cancer.

## Conclusion

The Amharic version of PHQ-9 appears to be a reliable and valid instrument to identify Major Depressive Episode among patients with chronic conditions such as cancer. Since it is free, brief, and easy to administer, this instrument can be used in resource-limited countries for depression screening.

## Data Availability

All data sets on which the conclusions of the manuscript will be available as spreadsheets documents upon request from the corresponding author.

## Abbreviations

AAU: Addis Ababa University
DSM-IV-IR: Diagnostic and Statistical Manual of Mental Disorders-IV text revision
ICD-10: International Classification of Diseases, 10th revision
MDE: Major Depressive Episode
MINI: Mini-International Neuropsychiatric Interview
PHQ-9: Patient Health Questionnaire − 9-item version
ROC: Receiver operating characteristic
TASH: Tikur Anbessa Specialized Hospital
